# Genetic diversity of *Plasmodium falciparum* populations in southeast and western Myanmar

**DOI:** 10.1186/s13071-017-2254-x

**Published:** 2017-07-04

**Authors:** Than Naing Soe, Yanrui Wu, Myo Win Tun, Xin Xu, Yue Hu, Yonghua Ruan, Aung Ye Naung Win, Myat Htut Nyunt, Nan Cho Nwe Mon, Kay Thwe Han, Khin Myo Aye, James Morris, Pincan Su, Zhaoqing Yang, Myat Phone Kyaw, Liwang Cui

**Affiliations:** 1 Department of Public Health, Ministry of Health and Sports, Nay Pyi Taw, Myanmar; 2 Department of Pathogen Biology and Immunology, Kunming Medical University, Kunming, China; 3 Department of Cell Biology & Genetics, Kunming Medical University, Kunming, China; 4 Department of Medical Research, Yangon city, Myanmar; 5 Department of Pathology, Kunming Medical University, Kunming, China; 6 Department of Genetics and Biochemistry, Eukaryotic Pathogens Innovation Center, Clemson University, Clemson, SC USA; 7 Transfusion Medicine Research Department, Yunnan Kunming Blood Center, Kunming, China; 8 Myanmar Medical Association, Yangon, Myanmar; 9 Department of Entomology, Pennsylvania State University, University Park city, PA 16802 USA

**Keywords:** Genetic diversity, *Plasmodium falciparum*, Multiclonal infection, Southeast and western Myanmar

## Abstract

**Background:**

The genetic diversity of malaria parasites reflects the complexity and size of the parasite populations. This study was designed to explore the genetic diversity of *Plasmodium falciparum* populations collected from two southeastern areas (Shwekyin and Myawaddy bordering Thailand) and one western area (Kyauktaw bordering Bangladesh) of Myanmar.

**Methods:**

A total of 267 blood samples collected from patients with acute *P. falciparum* infections during 2009 and 2010 were used for genotyping at the merozoite surface protein 1 (*Msp1*), *Msp2* and glutamate-rich protein (*Glurp*) loci.

**Results:**

One hundred and eighty four samples were successfully genotyped at three genes. The allelic distributions of the three genes were all significantly different among three areas. MAD20 and 3D7 were the most prevalent alleles in three areas for *Msp1* and *Msp2*, respectively. The *Glurp* allele with a bin size of 700–750 bp was the most prevalent both in Shwekyin and Myawaddy, whereas two alleles with bin sizes of 800–850 bp and 900–1000 bp were the most prevalent in the western site Kyauktaw. Overall, 73.91% of samples contained multiclonal infections, resulting in a mean multiplicity of infection (MOI) of 1.94. Interestingly, the MOI level presented a rising trend with the order of Myawaddy, Kyauktaw and Shwekyin, which also paralleled with the increasing frequencies of *Msp1* RO33 and *Msp2* FC27 200–250 bp alleles. *Msp1* and *Msp2* genes displayed higher levels of diversity and higher MOI rates than *Glurp*. PCR revealed four samples (two from Shwekyin and two from Myawaddy) with mixed infections of *P. falciparum* and *P. vivax*.

**Conclusions:**

This study genotyped parasite clinical samples from two southeast regions and one western state of Myanmar at the *Msp1*, *Msp2* and *Glurp* loci, which revealed high levels of genetic diversity and mixed-strain infections of *P. falciparum* populations at these sites. The results indicated that malaria transmission intensity in these regions remained high and more strengthened control efforts are needed. The genotypic data provided baseline information for monitoring the impacts of malaria elimination efforts in the region.

**Electronic supplementary material:**

The online version of this article (doi:10.1186/s13071-017-2254-x) contains supplementary material, which is available to authorized users.

## Background

Malaria caused by *P. falciparum* is one of the major human infectious diseases affecting millions of people worldwide. Despite enormous control efforts over several decades, malaria still remains an important public health problem. In 2015, there were about 214 million new malaria cases and 438,000 deaths [1]. In Southeast Asia, the Greater Mekong Subregion (GMS) has been one of the most malarious areas, with immense geographical heterogeneity in endemicity and the co-existence of different *Plasmodium* species [2, 3]. In this region, Myanmar has the greatest malaria burden. With enhanced control efforts by the national malaria control program and support from international partners, malaria in Myanmar has been steadily declining from 1341.8 cases per 100,000 population in 2005 to 253.3 in 2014 [4]. Yet, despite significant reduction of malaria incidence, *P. falciparum* parasites still maintained a high level of genetic diversity in northeastern Myanmar [5].

The genotypic and phenotypic diversity of malaria parasites enhances their ability to counteract control measures such as therapeutic drugs. In hyperendemic areas, each individual host may harbor multiple malaria parasite strains with different genotypes, including those that confer drug resistance [6]. Sexual reproduction involving malaria parasites of different genotypes, which can occur when a mosquito feeds on an individual infected with multiple parasite strains or when the same mosquito feeds on more than one human bearing distinct parasite genotypes, favors genetic recombination and generation of higher diversity. Thus, it is expected that parasite genetic diversity tends to correlate with the transmission intensity. Likewise, many studies have shown that malaria reduction as the result of intensified control efforts is accompanied by reduced genetic diversity of the parasite populations [7, 8] and in some cases leads to a clonal structure of the parasite population [9]. Notably, extensive use of certain antimalarial drugs can also alter the genetic diversity as a consequence of selective sweeps. Genetic diversity, malaria demographic history and other factors in a given region may reflect the transmission intensity, effectiveness of malaria control measures, and potential emergence of resistant parasites.

In this study, we assessed the genetic diversity of *P. falciparum* parasites from three areas in southeast and western Myanmar using three polymorphic markers: merozoite surface protein 1 (*Msp1*), *Msp2*, and the glutamate-rich protein (*Glurp*).

## Methods

### Collection of clinical parasite samples

**Fig. 1:**
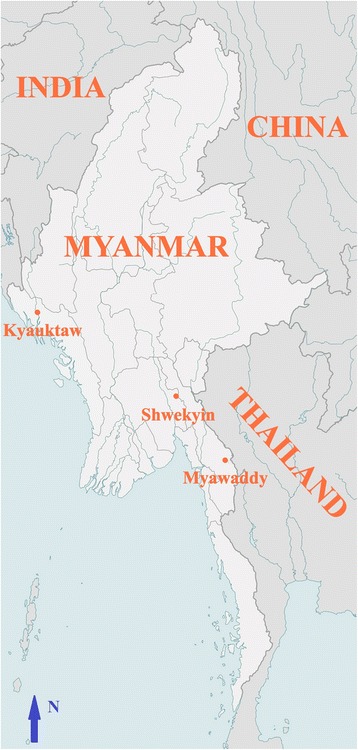
Map of Myanmar showing the sample collection sites

*Plasmodium falciparum* clinical samples were collected in 2009–2010 during efficacy studies of artemisinin combination therapies conducted in three locations in Myanmar (Shwekyin, Bago Division; Myawaddy, Kayin State; and Kyauktaw, Rakhine State; Fig. 1). Shwekyin and Myawaddy are located in southeast Myanmar, close to the Myanmar-Thailand border, whereas Kyauktaw is located in western Myanmar near the Myanmar-Bangladesh border. Shwekyin is ~510 km and ~220 km from Kyauktaw and Myawaddy, respectively. These sites were selected for the drug efficacy studies because they represent the malaria high-endemicity regions in Myanmar. In 2009, 112,653, 47,479 and 20,044 malaria cases were recorded in Rakhine State, Bago Division, and Kayin State, respectively. A total of 267 samples (56, 151, and 60 from Myawaddy, Shwekyin and Kyauktaw, respectively) were obtained from patients aged 6–70 years with acute malaria (Additional file 1: Table S1). All cases were confirmed to have *P. falciparum* infection by microscopic examination of Giemsa-stained thick and thin blood smears at the local clinics. For parasite genotyping, 200 μl of finger-pricked blood was spotted on 3 mm Whatman filter paper, dried, and stored at 4 °C until DNA extraction.

#### Species identification and genotyping of *P. falciparum Msp1, Msp2* and *Glurp*

Parasite genomic DNA was extracted from filter papers using the QIAamp DNA micro kit (Qiagen, Hilden, Germany) following the manufacturer’s instructions. Nested polymerase chain reaction (PCR) was used to identify the *Plasmodium* species with primers specific for *P. falciparum*, *P. vivax*, *P. malariae*, *P. ovale* and *P. knowlesi* [10–12]. The different genotypes of *Msp1*, *Msp2*, and *Glurp* genes were amplified by using a nested PCR method as previously described [5, 13, 14]. The PCR products were separated on 2% agarose gel and stained with ethidium bromide. The size of the PCR products was estimated based on their mobility relative to the standard DNA ladder marker (TaKaRa, Kusatsu, Japan).

### Estimating allele frequencies and clone numbers

Alleles were identified based on the type and the fragment size of PCR products and assigned to different size bins. If an isolate had one allele at each of the three loci, this sample was considered to have a monoclonal infection. The multiplicity of infection (MOI) was defined as the largest number of alleles among three loci detected in the sample. This measure is conservative and it likely underestimates the number of clones present [15]. Yet, this may best reflect the level of endemicity in these areas.

### Statistical analysis

The *Msp1, Msp2* and *Glurp* allele frequencies were expressed as percentages. The proportions of alleles observed in each gene among three areas were compared using the Chi-square test. Pairwise comparison of MOI was done by Student’s t-test. The correlation between MOI and percentages of gametocytemic patients was analyzed by using Spearman’s correlation. The *P* value less than 0.05 was considered statistically significant.

## Results

### Parasite samples

The demographic and clinical characteristics of the study populations are presented in Table 1. Since the samples were collected from drug efficacy trials and the number of patients at each site did not reflect the level of local malaria transmission, the data could not be used for vigorous epidemiological comparisons. Yet, a significantly higher density of asexual parasites was detected from children in Myawaddy. At the time of enrollment to the efficacy studies, 21.19%, 10.71% and 15% patients in Shwekyin, Myawaddy and Kyauktaw carried gametocytes, respectively (Table 1). Although all patients were confirmed to have *P. falciparum* infections by microscopic examination, nested PCR identified four samples as *P. falciparum* and *P. vivax* mixed infections (two from each of the southeast sites). Other *Plasmodium* species (*P. malariae, P. ovale* or *P. knowlesi*) were not detected.

**Table 1:** Comparison of patient profiles from three regions of Myanmar

	Shwekyin (Southeast Myanmar)	Myawaddy (Southeast Myanmar)	Kyauktaw (Western Myanmar)	Total
Number of patients (males %)	151 (72.85)	56 (67.86)	60 (75)	267 (72.28)
Age (yrs), median (range)	23 (6–66)	24 (6–56)	20 (6–56)	22 (6–66)
6–15 years, *n* (%)	32 (21.19)	19 (33.93)	27 (45)	78 (29.21)
> 15 years, *n* (%)	119 (78.81)	37 (66.07)	33 (55)	189 (70.79)
Feverish patients on day 0 (%) (axillary temperature ≥ 37.5 °C)	58.28	96.43	53.33	65.17
Temperature (°C), mean (range)	38.03 (36.10–41.40)	38.87 (36.80–40.00)	37.7 (36.40–40.20)	38.13 (36.10–41.40)
Asexual parasite density on day 0, geometric mean (range)				
6–15 years (*n* = 78)	18,430 (986–70,751)	21,686 (580–80,590)	16,126 (645–63,620)	18,425 (580– 80,590)
> 15 years (*n* = 189)	15,397 (504– 93,310)	8887 (681– 83,894)	13,494 (674– 56,369)	13,790 (504– 93,310)
Gametocytemic patients on day 0 (%)	21.19	10.71	15.00	17.23

### Variants of *Msp1, Msp2* and *Glurp* genes

Of the 267 samples, 184 were successfully genotyped in *Msp1*, *Msp2* and *Glurp* genes. The PCR fragment sizes and allelic frequencies for *Msp1*, *Msp2* and *Glurp* in three areas are shown in Table 2. The allelic distributions of *Msp1*, *Msp2* and *Glurp* genes were significantly different among three areas (Table 2). The most abundant allele types of *Msp1* and *Msp2* of all three areas were MAD20 and 3D7, respectively. Shwekyin and Myawaddy both shared the same dominant *Glurp* allele of 700–750 bp, but the most prevalent *Glurp* alleles in Kyauktaw were 800–850 bp and 900–1000 bp. Compared to *Glurp*, *Msp1* and *Msp2* displayed higher genetic diversity in all three areas (﻿Additional file 2: Table S2).

**Table 2:** Allele types and frequencies of *Msp1*, *Msp2* and *Glurp* genes

Gene (total allele numbers)	Type	Size (bp)	Shwekyin (Southeast Myanmar)	Myawaddy (Southeast Myanmar)	Kyauktaw (Western Myanmar)	Total	*P*-value
			*n* (%)	*n* (%)	*n* (%)		
*Msp1* (311)	K1	120–200	34 (19.54)	25 (34.73)	12 (18.46)	71 (22.83)	*χ* ^2^ = 21.03, *df* = 8 *P* = 0.0071
		250–300	0 (0)	1 (1.39)	3 (4.62)	4 (1.29)	
	MAD20	120–150	27 (15.52)	12 (16.67)	8 (12.31)	47 (15.11)	
		180–300	56 (32.18)	23 (31.94)	26 (40.00)	105 (33.76)	
	RO33	150–200	57 (32.76)	11 (15.28)	16 (24.62)	84 (27.01)	
*Msp2* (291)	3D7	350–400	14 (8.75)	19 (24.68)	1 (1.85)	34 (11.68)	*χ* ^2^ = 35.78, *df* = 10 *P* < 0.0001
		450–550	80 (50.00)	45 (58.44)	30 (55.56)	155 (53.26)	
		600–700	9 (5.63)	0 (0)	3 (5.56)	12 (4.12)	
	FC27	200–250	14 (8.75)	0 (0)	1 (1.85)	15 (5.15)	
		280–350	41 (25.63)	13 (16.88)	18 (33.33)	72 (24.74)	
		400–500	2 (1.25)	0 (0)	1 (1.85)	3 (1.03)	
*Glurp* (208)		500–600	10 (9.60)	1 (1.85)	0 (0)	11 (5.29)	*χ* ^2^ = 20.57, *df* = 6 *P* = 0.0022
		700–750	48 (46.20)	23 (42.6)	12 (24.0)	83 (39.9%)	
		800–850	28 (26.90)	20 (37.0)	19 (38.0)	67 (32.21%)	
		900–1000	18 (17.30)	10 (18.5)	19 (38.0)	47 (22.6%)	

### Multiclonal infections

Of the 184 samples successfully genotyped for all three genes, only 26.09% samples were mono- allelic at each of the three genes (Table 3). Analysis of *Msp1* and *Msp2* revealed 52.72% and 54.35% samples harboring multiclonal infections, respectively, whereas analysis of *Glurp* identified 13.04% multiclonal infections. When the genotyping results from three loci were combined, 73.91% of the samples were multiclonal infections, giving a mean MOI of 1.94. Among the three areas, the levels of multiclonal infections ranged from 64 to 80.21%, and the MOI ranged from 1.70 to 2.05. The lowest MOI was found in Myawaddy, which was significantly lower than that in Shwekyin (*t*-test: *t*_(144)_ = 3.096, *P* = 0.002). In addition, the MOI did not show significant correlation with the proportion of gametocytemic patients in each site (Spearman correlation: *r*_(184)_ = 0.1, *P* = 0.2).

**Table 3:** Multiclonal infections in Shwekyin, Myawaddy and Kyauktaw of Myanmar

	Number of alleles										
Genes^a^	Shwekyin (Southeast Myanmar, *n* = 96)				Myawaddy (Southeast Myanmar, *n* = 50)			Kyauktaw (Western Myanmar, *n* = 38)			Total (*n* = 184)
	2	3	4	Total	2	3	Total	2	3	Total	
*Msp1*	37	19	1	57 (59.38%)	20	1	21 (42.00%)	11	8	19 (50.00%)	97 (52.72%)
*Msp2*	56	4	0	60 (62.50%)	23	2	25 (50.00%)	14	1	15 (39.47%)	100 (54.35%)
*Glurp*	8	0	0	8 (8.33%)	4	0	4 (8.00%)	12	0	12 (31.58%)	24 (13.04%)
Combined	54	22	1	77 (80.21%)	29	3	32 (64.00%)	18	9	27 (71.05%)	136 (73.91%)
MOI (Mean ± SD)	2.05 ± 0.69				1.70 ± 0.58^b^			1.95 ± 0.73		1.94 ± 0.68	

^a^Number (%) of mixed strain infections identified based on one marker (*Msp1, Msp2* or *Glurp*) or three markers together (combined)

^b^MOI is significantly different between Myawaddy and Shwekyin (*t*_(144)_ = 3.096, *P* = 0.002)

## Discussion

The genetic diversity of *P. falciparum* parasites impacts malaria transmission and malaria control strategies. Therefore, it is important to resolve the genetic population structure of *P. falciparum* parasites in epidemic areas. Here we compared the genetic diversity of *P. falciparum* populations in two southeast regions and one western state of Myanmar using three polymorphic antigen markers. Consistent with earlier findings, genotyping the two *Msp* markers *Msp1* and *Msp2* identified significantly higher numbers of allele variants than genotyping *Glurp* [5, 17]. For *Msp1*, the MAD20 type was more prevalent than the RO33 and K1 alleles, as reported in other areas of the GMS including southwestern China [16], northeastern Myanmar [5] and the Thai-Myanmar border [17]. It was noteworthy that the frequency of the RO33 allele in the present study ranged from 15.28 to 32.76%, which is distinct from the rare presence of this type in parasites from the central Myanmar [18]. For *Msp2*, the 3D7 type was more abundant than the FC27 type, which was similar to what has been reported from the Thai-Myanmar border [17], but different from results in the northeastern Myanmar [5] and Laos [19]. Interestingly, all three sites shared the same most prevalent *Msp2* allele (3D7 type, allele size 450–550 bp). For *Glurp*, Shwekyin and Myawaddy both had the 700–750 bp allele as the most frequent, whereas the 800–850 bp and 900–1000 bp alleles were the most common ones from Kyauktaw. The genetic similarities of the analyzed *P. falciparum* may reflect the geographical proximity of the study areas and possible population mixing from neighboring areas of the GMS. For example, the genetic diversity of parasites from the southeast site Myawaddy described in this study was similar to that from the Thai-Myanmar border [17]. Overall, the three polymorphic markers revealed high levels of genetic diversity of parasite populations from the three areas. This suggests that despite the malaria control efforts, the parasite’s effective population remained large in these historically high-endemicity areas. Significant sharing of certain alleles may be attributed to extensive human migration along the border areas, which serves as a vehicle for introducing and spreading of parasites between sites [2].

The level of MOI usually reflects the degree of transmission intensity, although the association is not linear [7, 20]. Genotyping the three polymorphic markers in this study revealed that at least 73.91% of the analyzed samples were mixed infections. The largest number of alleles detected from a single isolate was 4, 3 and 2 for *Msp1*, *Msp2* and *Glurp*, respectively. Such a high level of mixed infections indicates that transmission intensity was still high in these regions. Moreover, the trend for increased MOI to parallel the increased frequencies of the *Msp1* RO33 and the *Msp2* FC27 200–250 bp alleles suggested that these allelic types may be used as molecular markers for malaria prevalence or burden assessment in the region. Whereas this study and our earlier work in northeastern Myanmar using the same antigenic markers identified similarly high levels of genetic diversity of the parasites in spite of recent drastic reduction in malaria cases, the northeastern region had significantly lower MOI [4]. As the genetic diversity is expected to decrease in the face of malaria elimination, the information provided herein may serve as the baseline data for continued epidemiological surveillance in these endemic regions.

It is interesting to note that although this study was not designed to detect the prevalence of all malaria parasites, PCR analysis did identify four cases of mixed *P*. *falciparum/ P. vivax* infections. These four misdiagnosed samples were not in the same area. Misdiagnosis of the mixed infections is most likely due to lower parasitemia normally associated with *P. vivax* infections. Given that the missed *P. vivax* co-infections would preclude treatment with primaquine for anti-relapse therapy, it is likely that some of these patients would experience *P. vivax* relapses. Therefore, more detailed epidemiological studies involving all human parasites are needed to clarify the prevalence, dynamics and genetics of the parasites in the endemic regions of Myanmar.

## Conclusion

This study described the genetic diversity of *P. falciparum* from two southeast regions and one western state of Myanmar during 2009–2010. Successful genotyping of 184 clinical samples at *Msp1*, *Msp2* and *Glurp* revealed high levels of genetic diversity and MOI, indicating that *P. falciparum* transmission remained relatively intense in these regions. These results can serve as the baseline information to monitor the effectiveness of the malaria elimination efforts. We can compare the MOI with future’s data, to assay the effect of the prevention measures.

## Additional files

Additional file 1: Table S1. The information of all samples. (XLS 376 kb)

Additional file 2: Table S2. Allele sizes and types of *Msp1*, *Msp2* and *Glurp* genes. (XLS 83 kb)

## Abbreviations

*Glurp*: glutamate-rich protein
GMS: Greater Mekong Subregion
MOI: multiplicity of infection
*Msp1*: merozoite surface protein 1
*Msp2*: merozoite surface protein 2
